# Nodular goiter with amyloid deposition in an elderly patient: fine-needle cytology diagnosis and review of the literature

**DOI:** 10.1186/1471-2482-13-S2-S43

**Published:** 2013-10-08

**Authors:** Vincenzo Di Crescenzo, Alfredo Garzi, Fara Petruzziello, Mariapia Cinelli, Lucio Catalano, Pio Zeppa, Mario Vitale

**Affiliations:** 1 Medicine and Surgery, University of Salerno, Baronissi, Salerno, Italy; 2 Pausilipon Hospital, Naples, Italy; 3 Public Health, University of Naples "Federico II", Naples, Italy; 4 Hematology, Biochemistry and Biomedical Biotechnology, University of Naples "Federico II", Naples, Italy

## Abstract

**Background:**

Amyloidosis is a systemic disease characterized by the extracellular deposition of amyloid fibrils in different organs and tissues. The thyroid gland may be affected by diffuse or nodular amyloid deposits, along with multiple myeloma (MM) (Amyloid Light-Chain Amyloidosis, AL amyloidosis) or chronic inflammatory diseases (Amyloid A Amyloidosis, AA amyloidosis), but thyroid gland involvement rarely appears as the first clinical manifestation in both conditions. The present study reports a case of primary thyroidal nodular amyloid goiter diagnosed by fine-needle cytology (FNC) in an elderly patient.

**Case report:**

A 66-year-old female patient presented with dysphagia and hoarseness; the patient suffered from rheumatoid arthritis but did not have kidney failure or altered thyroid function. Ultrasound examination (US) showed a 30 mm irregular, hypoechoic area in the left thyroid lobe. FNC showed abundant, dense and amorphous material similar to the one stained in purple at Diff-Quik stain and pinkish at the Papanicolaou. Spindle cells with thin, bland and bent nuclei were scattered in this material; few thyroid follicular cells were also present. An alcohol-fixed smear was stained with Congo red: the amyloid material appeared cherry red and it also showed apple-green birefringence when observed with a polarizing microscope. A differential diagnosis between different thyroid pathologies was considered and the cytological diagnosis of nodular amyloid goiter was pointed out. The patient underwent thyroid lobectomy and the subsequent histological examination confirmed the cytological diagnosis.

**Conclusions:**

FNC is a safe and effective procedure for the diagnosis of thyroid amyloidosis. Congo red-stained smears can be used to demonstrate the presence of amyloid material, showing the typical green birefringence under polarized light. An early and accurate cytological diagnosis may suggest an hematological screening and the appropriate treatment for the thyroid nodule.

## Background

Senescence and aging involving several mechanisms like oxidative stress and elevated ROS (Reactive oxygen species). They has been implicated in cancer, diabetes, neurodegenerative, cardiovascular and other diseases [1,2]. Several stressors, including high-caloric diets, physical activity, chemicals, drugs and pollutants, induce oxidants overproduction [3]. Amyloidosis includes different forms characterized by the extracellular accumulation of insoluble, antiparallel β-pleated sheets of fibrils of proteins in different tissues and organs [4]. Amyloidosis is traditionally classified as Primary Amyloidosis (PA), arising from plasma cells diseases such as multiple myeloma (MM) [5] or other immunocyte dyscrasias, and Secondary Amyloidosis (SA) caused by a variety of degenerative, metabolic and inflammatory diseases [6-12]. Amyloidosis may involve different organs with different clinical manifestations related to the corresponding functions. Different types of human proteins have been identified as possible causative agents of amyloidosis [13], including amyloid light chain, SAA, β amyloid/APP and transthyretin [14]. Despite the different etiologies, organs involved, clinical manifestations and variety of proteins that can cause amyloidosis, a common feature is the accumulation of insoluble proteins arranged in cross-β-pleated sheet structures regardless of their source, primary structure or function [8]. Amyloid goiter (AG) is a rare condition characterized by thyroid infiltration of amyloid material, which causes thyroid gland enlargement and atrophy of thyroid follicles [15-18]. The most commonly reported clinical features of these patients are rapid, painless thyroid gland enlargement that may be associated with dysphagia, dyspnea, or hoarseness [15,16]. AG has been infrequently described [16,18] and most of the reported cases mainly refers to patients suffering from systemic amyloid A (AA) amyloidosis or long-standing predisposing diseases [19,20]. Palpable neck masses are not a rare occurrence, some time representing a challenging diagnostic dilemma with unusual extrathyroidal masses [21,22]. Fine-needle cytology (FNC) is a primary diagnostic tool in preoperative diagnosis of thyroid nodules [23-28]. Cellular biomarkers, such as endothelial progenitor cells, whose frequency increase in peripheral blood of cancer patients and decrease in those suffering from cardiovascular diseases [29-31], are unfortunately lacking. However, the application of immunocytochemistry (ICC), flow cytometry (FC) and molecular techniques to FNC has dramatically increased the sensitivity of the method [28,32-38]. The identification of chromosomal aberrations or differences in the expression profiles of suitable membrane ion channels, such as ion channels, whose expression may be up-regulated under pathological conditions [39-43], might favour amyloidosis recognition. These advantages are enhanced in case of AG, which does not require surgical treatment, and even more in elderly patients, for whom surgery is generally more burdensome, complex and expensive than younger patients [44-46]. A case of nodular AG diagnosed by FNC is here described; differential diagnosis and clinical implication of the FNC diagnosis are described accordingly.

## Case report

A 66-year-old female with a palpable thyroid nodule, complaining of dysphagia and hoarseness, was admitted to the outpatient endocrinology clinics of the Azienda Ospedaliera Universitaria, University of Salerno. The patient suffered from a light form of rheumatoid arthritis and did not have kidney failure. She also complained of painless thyroid gland enlargement in the previous 5 months. Serum levels of thyroid hormones and TSH were in the normal range, including calcitonin. Ultrasound examination showed a 30 mm large, irregular in shape, hypoechoic area in the left thyroid lobe. The remaining gland was normal in size and shape. No relevant lymph nodes were detected. The patient underwent US-guided FNC with rapid on-site evaluation (ROSE), as previously described [47,48]. The diagnostic procedure and its related risks were first discussed with the patient, who was informed that 1 or 2 additional passes might have been needed, and an informed consent was obtained. The first smear was immediately Diff-Quik stained and evaluated on site. Two additional passes were used to prepare four alcohol-fixed smears that were subsequently used for Papanicoaou, Congo red stain and immunocytochemistry using thyroglobulin antibody. Technical details for both procedures have been previously described [32,49,50].

## Results

**Figure 1:**
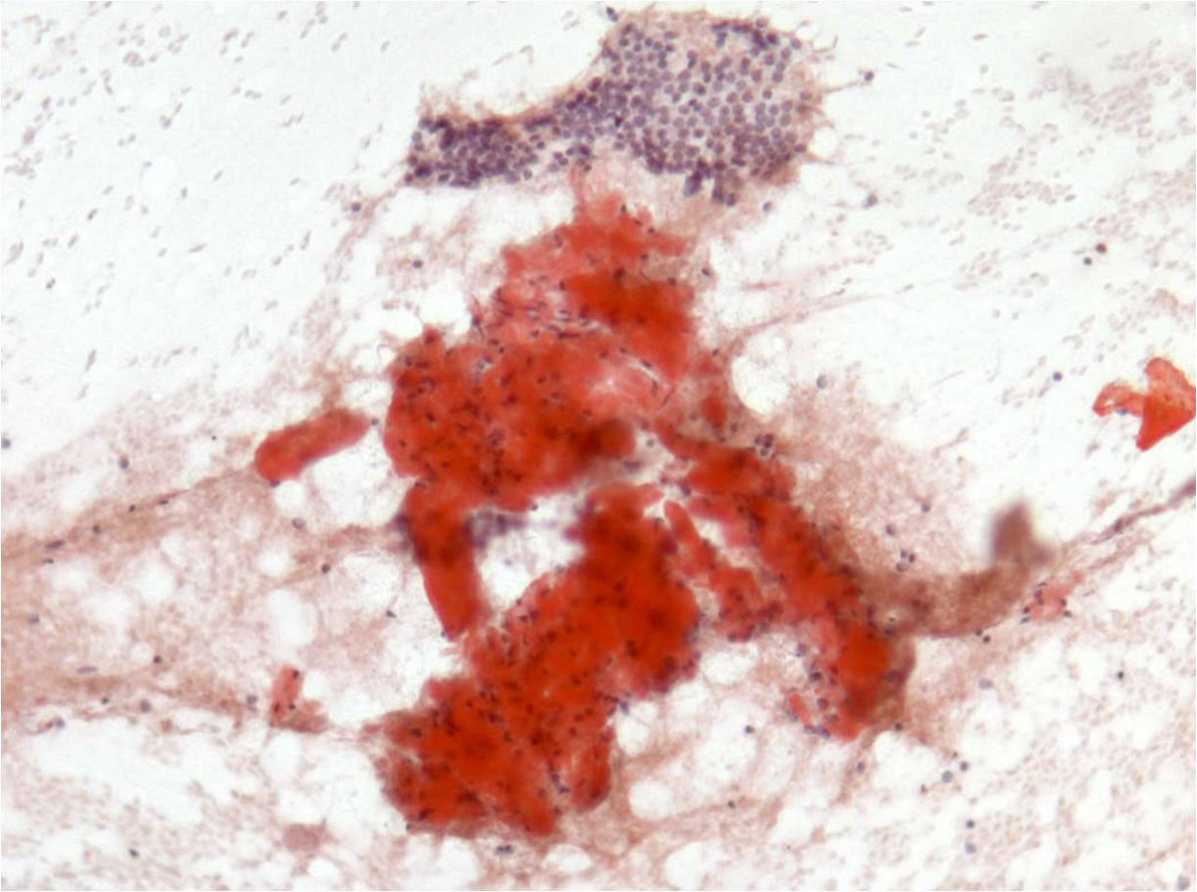
**Abundant amorphous amyloid material appearing solid dark-violet blue on the Diff-Quik stain (Diff-Quik stain 103 X)**.

**Figure 2:**
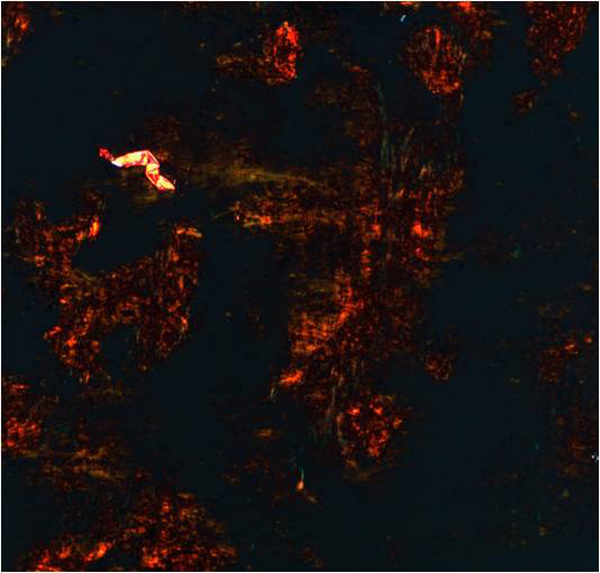
**Amyloid deposit, Congo red stained, as it appears under polarized light: red with apple-green birefringence**.

The smears were moderately cellular with the presence of abundant amorphous material interspersed among few follicular cells (Figure 1). This material was more solid and hyaline than the colloid and appeared dark-violet blue on the Diff-Quik and pinkish-orange on the Papanicolaou-stained smears respectively. Numerous spindle cells were interspersed in this amorphous material. These cells were mainly isolated and showed small spindle and bent nuclei with dense compact chromatin without nucleoli and showed lack of cytoplasm. The few follicular cells were present in small groups at the edges of this material and showed occasional cytoplasmic vacuolation with paravacuolar granulations. Because of these cytological features, one of the alcohol-fixed smear was stained with Congo red using an appropriate positive control. The smear showed an amorphous material which appeared dense, orange and brilliant and showed a metachromatic green under the polarized microscope (Figure 2). Follicular cells showed cytoplasmic positivity at the immunostain for thyroglobulin, performed on the additional smear assessing the thyroid origin.

## Discussion

Amyloidosis refers to a variety of conditions in which insoluble proteins are deposited in the extracellular space of various organs or tissues. Approximately 60 different proteins have been identified, giving the name to the different clinical forms prefixed with the letter A [9,14]. Therefore, amyloidosis is termed AL amyloidosis in case of overproduction of immunoglobulin light chains in MM, and AA amyloidosis in case of continuous acute phase protein production, as in chronic inflammations. AL amyloidosis is caused by tissue-infiltrating proteins from monoclonal immunoglobulin light chains. AL amyloid is made of proteins arranged in cross- β -pleated sheet structures [4]. Fibrils are usually formed by N-terminal fragments of the variable region of an immunoglobulin light chain, although they may occasionally include part of a constant region or may be formed by whole light chain [5,6]. Only a small portion of the light chain is able to form amyloid fibrils: this property is likely correlated with the distinctive structural features of amyloidogenic light chains, as almost 70% of them are λ isotype and the V λ 6a and V λ 3r genes encode nearly 40% of these chains [7]. Amyloidosis is classified as 'localized' when it affects one body organ or tissue type and 'systemic' when more than one organ or system are involved; it was also classified as 'primary' when related to disorders of immunocompetent cells such as MM or other plasma cells dyscrasias and 'secondary' or 'reactive' when occurring as a complication of chronic degenerative, inflammatory infective or autoimmune diseases [4,7,8,12-14]. Clinical relevance and prognosis of amyloidosis largely depend on the organ involved. In case of AG, the prognosis mostly depends on the primary disease and the possible involvement of other organs [4]. In case of sole thyroid localization, prognosis is reported to be relatively favorable and conservative treatment or limited surgery is recommended. As for FNC diagnosis, because of the rapidly growing mass and the presence of spindle cells in the smears, the differential diagnosis of different thyroid and extra-thyroid benign and malignant entities (i.e. mesenchymal tumors of the neck such as pseudosarcomatous lesions, fibrosarcoma and other mesenchymal malignancies) was pointed out [51-56]. However these lesions were excluded because of the bland appearance of the above spindle cells and because of the amyloid material that was clearly different from the fibrous stroma fragments that may be observed in mesenchymal tumors. Conversely, because of the presence of amyloid material and spindle cells, a possible spindle cell medullary thyroid carcinoma was considered. The bland appearance of the nuclei and the normal serum levels of calcitonin ruled out this possibility. Finally, the optical properties of amyloid material, the presence of few benign follicular cells and the small number of spindle cells favored the diagnosis of AG. On the basis of the cytological diagnosis, a conservative surgery was performed and the histological analysis confirmed the FNC diagnosis of AG. In conclusion, although rare, AG may be diagnosed by FNC, provided that the amyloid nature of accumulated material is identified. The cytological diagnosis may be useful for a timely screening for systemic amyloidosis and for an adequate treatment of the thyroid localization avoiding useless extensive surgical treatments.

## Conclusions

FNC is a safe and effective procedure for the diagnosis of thyroid amyloidosis. Congo red-stained smears can be used to demonstrate the presence of amyloid material, showing the typical green birefringence under polarized light. An early and accurate cytological diagnosis may suggest an hematological screening and the appropriate treatment for the thyroid nodule.

## Competing interests

The authors declare that they have no competing interests.

## Authors' contributions

MV: conception and design, interpretation of data, AG, PZ, VDS, AG, LC, MPC: acquisition of data, drafting the manuscript, PZ, MV: critical revision, given final approval of the version to be published.

## Authors' information

VDC = Aggregate Professor of Thoracic Surgery at University of Salerno

AG = Aggregate Professor of Pediatric Surgery at University of Salerno

FP= Assistant of Hematology at Pausilipon Hospital, Naples

MC = Aggregate Professor of Anatomy, University of Naples "Federico II"

LC= Assistant of Hematology at University of Naples "Federico II"

PZ = Associate Professor of Pathology at University of Salerno

MV = Associate Professor of Endocrinology at University of Salerno

## Declarations

Funding for this article came from University funds.

This article has been published as part of *BMC Surgery *Volume 13 Supplement 2, 2013: Proceedings from the 26th National Congress of the Italian Society of Geriatric Surgery. The full contents of the supplement are available online at http://www.biomedcentral.com/bmcsurg/supplements/13/S2
